# Supplementary material for: Finding high posterior density phylogenies by systematically extending a directed acyclic graph
Source: ArXiv. 2024 Nov 18:arXiv:2411.09074v2. Preprint. [Version 2] (PMC11601806)
Supplement: Supplement 1 [file NIHPP2411.09074v2-supplement-1.pdf]

Supplementary Materials

Additional Benchmarking Figures

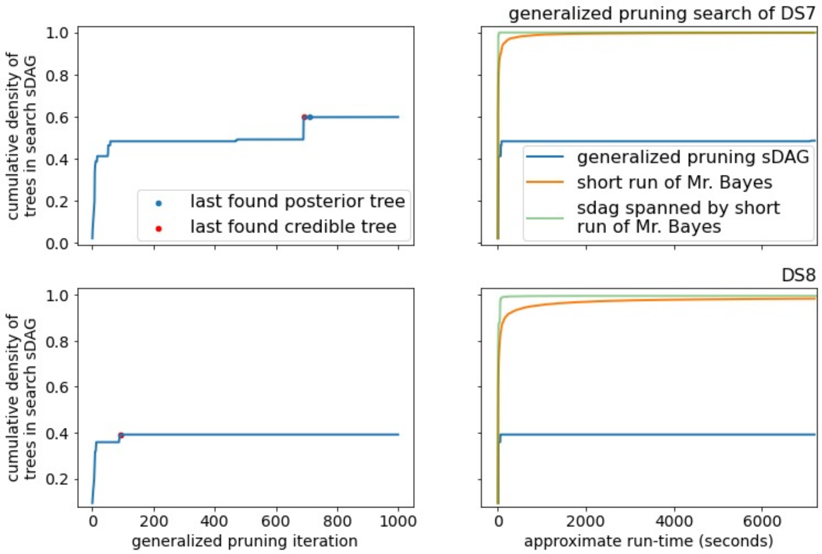

Figure S1: The empirical posterior density found by generalized pruning on the remaining DS-datasets and a comparison with MCMC.

Compatible Subsplits and Edges

At the end of the subsection on Performing NNIs to the subsplit DAG, we stated our preference for sDAGs with edges between all compatible subsplits and some properties of such sDAGs. Having described the top pruning algorithm, we now explain these preferences. Consider the likelihood in (1), for which the top pruning likelihood serves as a proxy. For an sDAG without edges between all compatible subsplits, we would need to consider topologies past those that contain the central edge. It is possible for a new topology to exist in the post-NNI sDAG, not contain the central edge, yet have the maximum likelihood. Even among topologies containing the central edge, the one of maximum likelihood may not be an NNI of a topology in the pre-NNI sDAG, making it impossible for a best known tree to obtain the maximum likelihood. Maintaining an sDAG with all possible edges prevents these issues.

Next we provide a proof of those properties and an example of an sDAG with missing edges without those properties. Recall by "compatible subsplits", we mean two subsplits t and s such that s bipartitions one of the subsplit-clades of t, and an sDAG is missing an edge if it contains compatible subsplits t and s but not the edge $t \to s$.

Proposition. *Suppose $\mathcal{D}$ is an sDAG with edges between all compatible subsplits, $\mathcal{D}'$ is an sDAG given by applying an NNI to $\mathcal{D}$, and $t' \to s'$ in $\mathcal{D}'$ is the*

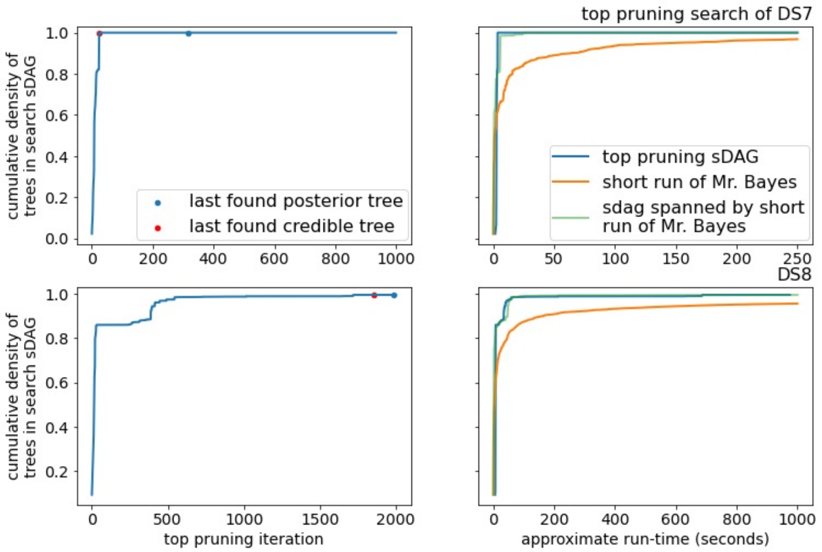

Figure S2: The empirical posterior density found by top pruning on the remaining DS-datasets and a comparison with MCMC. Note the x-axis scale varies between data sets.

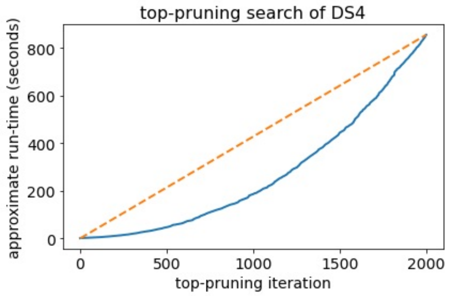

Figure S3: The run-time (blue) of top pruning on DS4. The straight dotted orange line visually confirms convexity. All other data sets exhibit similar run-time behavior.

central edge of the NNI. If a topology τ' is in $\mathcal{D}'$ and not in $\mathcal{D}$, then the edge $t' \to s'$ is in τ' and there exists a topology τ in $\mathcal{D}$ such that τ' is an NNI of τ.

Proof. First we recall some facts about NNIs on sDAGs that follow directly from the definition. There are at most two subsplits in $\mathcal{D}'$ that are not in $\mathcal{D}$ (if such subsplits exist, they are among t' and s'). There is at most one edge that is in $\mathcal{D}'$, is not in $\mathcal{D}$, and is of the form $t' \to s^*$ with $\bigcup(s^*) = \bigcup(s')$ (if such an edge exists, it is $t' \to s'$). There is at most one edge that is in $\mathcal{D}'$, is not in $\mathcal{D}$, and is of the form $t^* \to s'$ (if such an edge exists, it is $t' \to s'$). Additionally,

since $\mathcal{D}$ has all compatible edges, we can verify a generic edge $t^* \to s^*$ is in $\mathcal{D}$ by showing only that t^* and s^* are in $\mathcal{D}$. Similarly, we can verify a generic topology τ^* is in $\mathcal{D}$ by showing only that each subsplit in τ^* is in $\mathcal{D}$. Note τ' is a topology and so it also has all compatible edges.

First we prove that $t' \to s'$ is in τ' by showing t' and s' are in τ'. At least one of t' and s' must be in τ', as otherwise all subsplits of τ' are in $\mathcal{D}$ and so τ' is in $\mathcal{D}$.

Suppose t' is in τ'. As t' is not a leaf, there is some edge $t' \to s^*$ in τ' with $\bigcup(s^*) = \bigcup(s')$. We have a contradiction if $s^* \neq s'$, as $s^* \neq s'$ implies $t' \to s^*$ is in $\mathcal{D}$, meaning all subsplits of τ' are in $\mathcal{D}$ and so τ' is in $\mathcal{D}$. Thus $s^* = s'$, so s' is in τ'.

Suppose s' is in τ'. As s' is not the root, there is some edge $t^* \to s'$ in τ'. We have a contradiction if $t^* \neq t'$, as $t^* \neq t'$ implies $t^* \to s'$ is in $\mathcal{D}$, meaning all subsplits of τ' are in $\mathcal{D}$. Thus $t^* = t'$, so t' is in τ'.

Thus both t' and s' are subsplits of τ', and so $t' \to s'$ is in τ'. To obtain τ from τ', we apply the appropriate NNI to τ' at $t' \to s'$. Specifically, suppose the subsplits and edges in τ' near $t' \to s'$ are $u \to t'$, $t' \to y$, $s' \to x$, and $s' \to z$. Further suppose the NNI enlarging $\mathcal{D}$ to $\mathcal{D}'$ swapped $\bigcup(y)$ with $\bigcup(z)$ at $t \to s$ (the case of y with x is identical). Let τ by the topology given by τ' after removing t' and s', removing $t' \to s'$ and the neighboring four edges, adding the subsplits t and s, and adding the edges $u \to t$, $t \to s$, $t \to z$, $s \to x$, and $s \to y$. All of these edges are between compatible subsplits of $\mathcal{D}$ and so τ is in $\mathcal{D}$. Since τ is an NNI of τ', τ' is an NNI of τ. $\square$

To see how issues may arise when an sDAG is missing edges between compatible subsplits, consider the three topologies:

$$(0, (((((1, (2, 3)), (4, 5)), (6, (7, 8))), 9), 10)),$$
$$(0, (((1, ((2, 6), ((3, 7), 8))), (4, 5)), (9, 10))), \text{ and}$$
$$(0, ((((((((1, 2), 3)), ((6, 7), 8)), 4), 5), 10), 9)).$$

Let $\mathcal{D}$ be the sDAG generated by these topologies. A series of lengthy, but trivial calculations, shows that

- $\mathcal{D}$ contains only the three input topologies;
- $\mathcal{D}$ is missing edges between compatible subsplits;
- both the topology $(0, (((((1, 2), 3), ((6, 7), 8)), (4, 5)), (9, 10)))$ and the topology $(0, ((((1, ((2, 6), ((3, 7), 8))), (4, 5)), 9), 10))$ are in $\mathcal{D}'$, the sDAG obtained by enlarging $\mathcal{D}$ with the NNI swapping $\{4, 5\}$ and $\{6, 7, 8\}$ at the edge $\{\{1, 2, 3, 4, 5\}, \{6, 7, 8\}\} \to \{\{1, 2, 3\}, \{4, 5\}\}$;
- the topology $(0, (((((1, 2), 3), ((6, 7), 8)), (4, 5)), (9, 10)))$ is not an NNI of any topology in $\mathcal{D}$;
- and the topology $(0, ((((1, ((2, 6), ((3, 7), 8))), (4, 5)), 9), 10))$ does not contain the central edge $\{\{1, 2, 3, 6, 7, 8\}, \{4, 5\}\} \to \{\{1, 2, 3\}, \{6, 7, 8\}\}$.

Initialization of Choice Maps

Here we formalize the concept of edge choice maps and best known tree used in the top pruning algorithm. Suppose we have a list of trees, the phylogenetic likelihoods of these trees, and the sDAG constructed from the trees. We define a rootward choice map as a map from each edge of the sDAG to the sibling and parent edges taken from the maximum likelihood input tree containing the edge. Similarly, we define a leafward choice map as a map from each edge of the sDAG to the two child edges taken from the maximum likelihood input tree containing the edge. Note these choices are made at the level of sDAG edges, not subsplits. We also store branch lengths for every sDAG edge, where an edge of the sDAG takes its branch lengths from the maximum likelihood input tree that contains the edge.

Given these maps we apply them recursively given a starting edge, filling out a topology. Since the edges also have assigned branch lengths, we have a tree that is ready for likelihood evaluation. For a given edge, the tree constructed from the choice maps and branch lengths is what we take as the *best known tree* containing the edge. With multiple input trees, the best known tree for an edge produced by the choice maps need not be one of the input trees, as seen in Figure 5.

We use the so-called best known tree for an edge instead of the maximum likelihood tree containing the edge because the latter is not computationally feasible. Consider an edge from a parent subsplit t to a child subsplit s. Intuitively, we can construct all topologies in the sDAG that contain the edge $t \to s$ by choosing neighboring edges and moving outward until we have constructed a full topology. We would explore all combinations of an edge ending in the parent t, an edge leaving the parent t to a subsplit bipartitioning the clade $\bigcup(t) - \bigcup(s)$, an edge leaving the child s to a subsplit bipartitioning one subsplit-clade of s, and an edge leaving the child s to a subsplit bipartitioning the other subsplit-clade. We then continue outward exploring all options of neighbors for the new edges. This is depicted in Figure S4. Furthermore, we would need to calculate the likelihoods of all of these trees. Our best known trees greatly restrict how often we choose neighboring edges. In particular, for each edge of the sDAG we make the choice of four neighbors only once.

At this step, we have defined choice maps and best known trees for edges in an sDAG generated from a specific list of trees. In the next subsection, we explain how we maintain these choice maps as we grow an sDAG with NNIs.

Maintaining Choice Maps

Suppose we are in the situation where we have an sDAG $\mathcal{D}$ with fully defined choice maps and branch lengths. For an NNI on $\mathcal{D}$ producing $\mathcal{D}'$, we must define the choice maps on the edges of $\mathcal{D}'$. For edges common to $\mathcal{D}$ and $\mathcal{D}'$, we use the choice maps of $\mathcal{D}$. The remaining edges are of the five types discussed in the subsection Performing NNIs to the subsplit DAG. Suppose the sDAGs and edges are as depicted in Figure S5. Let u, x, y, and z denote the subsplits of $\mathcal{D}$

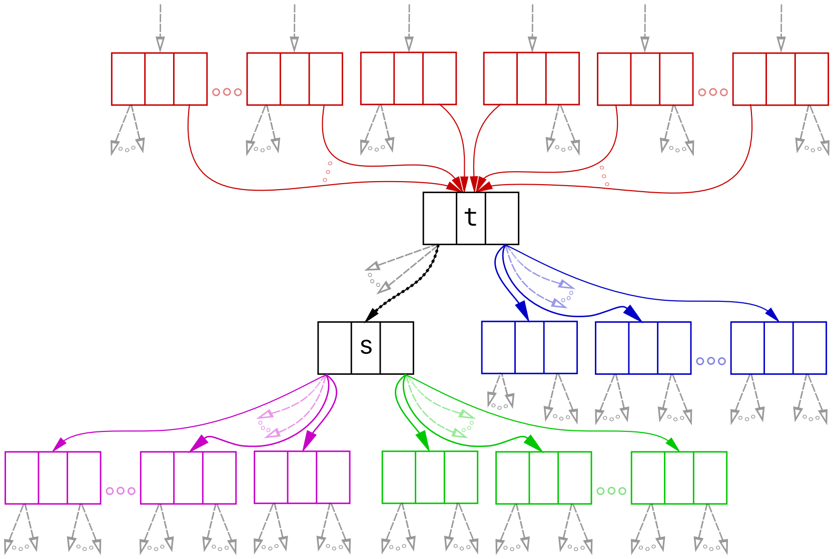

Figure S4: The sDAG structure near the edge $t \to s$, which is selected to start. The parent subsplits of t in the sDAG are in red; the child subsplits of t opposite s are in blue; the child subsplits of s bipartitioning one subsplit clade of s are in green; and the child subsplits of s bipartitioning the other subsplit clade of s are in purple. Constructing all topologies with the edge $t \to s$ begins by taking all combinations of selecting one of each of the red, blue, green, and purple subsplits and edges.

where $u \to t$, $s \to x$, $s \to y$, and $t \to z$ are the parent, left child, right child, and sibling edges given by the choice maps at $t \to s$. Let u^*, x^*, y^*, and z^* denote arbitrary subsplits with $u^* \to t$, $s \to x^*$, $s \to y^*$, and $t \to z^* \in \mathcal{D}$.

We define choice maps for edges with these subsplits, t', and s' (where $t' \to s'$ is the central edge of the NNI) as follows:

$$\begin{aligned}
&\text{parent}(u^* \to t') = \text{parent}(u^* \to t), && \text{sibling}(u^* \to t') = \text{sibling}(u^* \to t), \quad (3)\\
&\text{child}_1(u^* \to t') = t' \to s', && \text{child}_2(u^* \to t') = t' \to y,\\
&\text{branch_length}(u^* \to t') = \text{branch_length}(u^* \to t), &&\\
&\text{parent}(t' \to s') = u \to t', && \text{sibling}(t' \to s') = t' \to y,\\
&\text{child}_1(t' \to s') = s' \to x, && \text{child}_2(t' \to s') = s' \to z,\\
&\text{branch_length}(t' \to s') = \text{branch_length}(t \to s), &&\\
&\text{parent}(t' \to y^*) = u \to t', && \text{sibling}(t' \to y^*) = t' \to s',\\
&\text{child}_1(t' \to y^*) = \text{child}_1(s \to y^*), && \text{child}_2(t' \to y^*) = \text{child}_2(s \to y^*),\\
&\text{branch_length}(t' \to y^*) = \text{branch_length}(s \to y^*), &&\\
&\text{parent}(s' \to x^*) = t' \to s', && \text{sibling}(s' \to x^*) = s' \to z,\\
&\text{child}_1(s' \to x^*) = \text{child}_1(s \to x^*), && \text{child}_2(s' \to x^*) = \text{child}_2(s \to x^*),
\end{aligned}$$

$$
\begin{aligned}
&\text{branch\_length}(s' \to x^*) = \text{branch\_length}(s \to x^*), \\
&\text{parent}(s' \to z^*) = t' \to s', \qquad \text{sibling}(s' \to z^*) = s' \to x, \\
&\text{child}_1(s' \to z^*) = \text{child}_1(t \to z^*), \qquad \text{child}_2(s' \to z^*) = \text{child}_2(t \to z^*), \\
&\text{branch\_length}(s' \to z^*) = \text{branch\_length}(t \to z^*).
\end{aligned}
$$

We emphasize that for edges common to $\mathcal{D}$ and $\mathcal{D}'$, we use the values from $\mathcal{D}$, not those in equation (3).

In summary, given a pre-NNI sDAG with defined choice maps, we extend the choice maps to the post-NNI sDAG. For the branch lengths, we use those in equation (3) as starting values for optimization. We optimize these branch lengths to maximize the standard phylogentic likelihood of the best known tree associated to each edge. For edges present in $\mathcal{D}$, the branch lengths are unaltered. The edge $t' \to s'$ of $\mathcal{D}'$ has a well-defined best known tree. We define the top pruning likelihood of the NNI to be the likelihood of this tree.

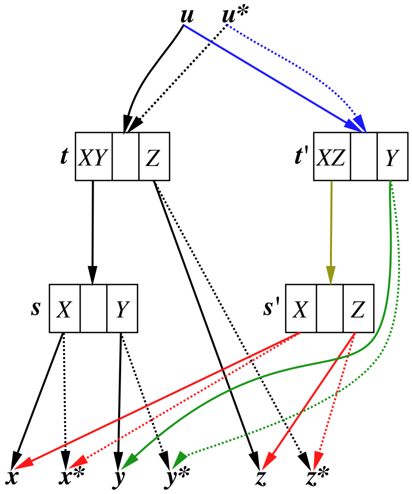

Figure S5: Subsplits and edges for the NNI swapping clades Y and Z at the edge $t \to s$. The potentially new subsplits are t' and s'. Edges in black are existing edges of the sDAG, with solid lines indicating edges selected by the choice maps at $t \to s$ and dotted lines are additional edges. Specifically, the existing choice maps take parent$(t \to s) = u \to t$, sibling$(t \to s) = t \to z$, child$_1(t \to s) = s \to x$, and child$_2(t \to s) = s \to y$. Edges in color are potentially new edges, with solid lines being edges selected by choice maps at new edges and dotted lines are additional edges.

To maintain an sDAG with edges between all compatible subsplits, we require choice maps and branch lengths at two additional types of edges. These edges take the either the form $t' \to s^*$, with $\bigcup(s^*) = \bigcup(s')$, or $t^* \to s'$. Denote the left subsplit-clade of s^* by X^*, the right subsplit-clade of s^* by Z^*, and the subsplit-clade opposite s' of t^* by Y^*. The choice maps for such edges are given

by,

$$
\begin{aligned}
\text{parent}(t' \to s^*) &= \text{parent}(t' \to s'),\\
\text{sibling}(t' \to s^*) &= \text{sibling}(t' \to s'),\\
\text{child}_1(t' \to s^*) &= \operatorname*{argmax}_{\substack{s^* \to x^* \in \mathcal{D},\\ \bigcup(x^*)=X^*}} p_\psi(\mathbf{Y} \mid \mathcal{B}(s^* \to x^*)),\\
\text{child}_2(t' \to s^*) &= \operatorname*{argmax}_{\substack{s^* \to z^* \in \mathcal{D},\\ \bigcup(z^*)=Z^*}} p_\psi(\mathbf{Y} \mid \mathcal{B}(s^* \to z^*)),\\
\text{parent}(t^* \to s') &= \operatorname*{argmax}_{u^* \to t^* \in \mathcal{D}} p_\psi(\mathbf{Y} \mid \mathcal{B}(u^* \to t^*)),\\
\text{sibling}(t^* \to s') &= \operatorname*{argmax}_{\substack{t^* \to y^* \in \mathcal{D},\\ \bigcup(y^*)=Y^*}} p_\psi(\mathbf{Y} \mid \mathcal{B}(t^* \to y^*)),\\
\text{child}_1(t^* \to s') &= \text{child}_1(t' \to s'),\\
\text{child}_2(t^* \to s') &= \text{child}_2(t' \to s'),
\end{aligned}
\tag{4}
$$

where $\mathcal{B}(e)$ denotes the best known tree for the edge e. With the choice maps defined, we then assign branch lengths to $t' \to s^*$ and $t^* \to s'$ to maximize the phylogenetic likelihood of the best known trees for these edges. This allows us to extend choice maps and branch lengths of $\mathcal{D}$ to those for an sDAG obtained by an NNI and adding all compatible edges.

How the sDAG Captures the Posterior

Thinking in a more general sense, we can ask the question, "does the sDAG help us find additional trees in the topological posterior distribution?" With top pruning, the answer is yes, but what about building the sDAG from a collection of trees sampled from the posterior? (Note in this section, in contrast to the main body of the paper, we do not add all compatible edges.) Necessarily the posterior density of topologies in an sDAG is at least that of the topologies used to construct the sDAG. We also know that the number of topologies in an sDAG grows very fast with the number of input topologies. But how much is the additional posterior density and how many additional credible topologies are in the sDAG?

Starting from the beginning of an MCMC run on the data set, let S be the set of topologies sampled after k MCMC generations, $\mathcal{D}$ be the sDAG built from S, and T be the set of topologies in $\mathcal{D}$. How large are: the posterior density of trees in S, the posterior density of trees in T, and $S \cap C$ relative to $T \cap C$, where C is the 95% credible set? Rather than plotting these values against k, the number of generations, we use the number of distinct topologies found after k generations.

Let us examine these plots (Figure S6) in terms of posterior density, S, T, and C. For posterior density, the gain in using an sDAG built from the input trees is seen by comparing the blue lines to the orange lines. These gains vary by data set, with the more diffuse posteriors yielding higher gains.

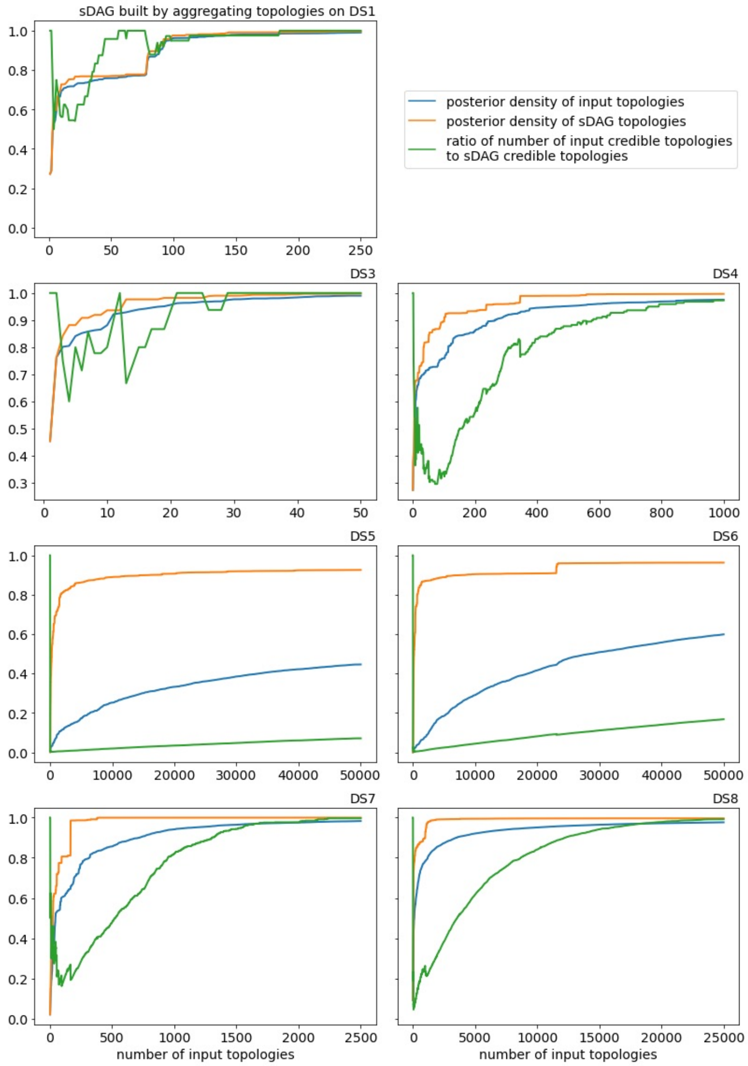

Figure S6: Topologies and spanned sDAGs from MCMC exploration of the DS-datasets. Input topologies are from the short runs of `MrBayes` described earlier.

The green lines, which relate to the credible set C, are harder to interpret. All plots begin in the top-left corner, because the short MCMC runs start at the maximum posterior tree (i.e., the first topology is credible). The behavior past that is again dependent on how diffuse is the posterior distribution. For DS1 and DS3 (the least diffuse), the sDAG initially provides additional credible topologies, but these topologies are quickly found by the short MCMC run. For DS5 and DS6 (the most diffuse), the sDAG provides a large number of credible topologies not found by the short MCMC run. On the remaining data

sets, which are DS4, DS7, and DS8, the extent to which the sDAG contains additional credible topologies follows the ranking of how diffuse these data sets are.

Overall, the pattern is that for a diffuse data set, taking reasonable topologies and forming an sDAG could be very advantageous. However, if the posterior is rather compact, then the sDAG gives some additional information but at costly price in terms of the number of topologies.
